# Analysis of Research Activity in Gastroenterology: Pancreatitis Is in Real Danger

**DOI:** 10.1371/journal.pone.0165244

**Published:** 2016-10-24

**Authors:** Andrea Szentesi, Emese Tóth, Emese Bálint, Júlia Fanczal, Tamara Madácsy, Dorottya Laczkó, Imre Ignáth, Anita Balázs, Petra Pallagi, József Maléth, Zoltán Rakonczay, Balázs Kui, Dóra Illés, Katalin Márta, Ágnes Blaskó, Alexandra Demcsák, Andrea Párniczky, Gabriella Pár, Szilárd Gódi, Dóra Mosztbacher, Ákos Szücs, Adrienn Halász, Ferenc Izbéki, Nelli Farkas, Péter Hegyi, Hungarian Pancreatic Study Group

**Affiliations:** 1 Institute for Translational Medicine, University of Pécs, Pécs, Hungary; 2 First Department of Medicine, University of Szeged, Szeged, Hungary; 3 Department of Pathophysiology, University of Szeged, Szeged, Hungary; 4 Department of Pediatrics and Pediatric Health Center, University of Szeged, Szeged, Hungary; 5 Heim Pál Children’s Hospital, Budapest, Hungary; 6 Department of Gastroenterology, First Department of Medicine, University of Pécs, Pécs, Hungary; 7 Department of Translational Medicine, First Department of Medicine, University of Pécs, Pécs, Hungary; 8 Department of Pediatrics, János Balassa Hospital of County Tolna, Szekszárd, Hungary; 9 First Department of Surgery, Semmelweis University, Budapest, Hungary; 10 First Department of Medicine, St. George University Teaching Hospital of County Fejér, Székesfehérvár, Hungary; 11 Institute of Bioanalysis, University of Pécs, Pécs, Hungary; 12 Hungarian Academy of Sciences—University of Szeged, Momentum Gastroenterology Multidisciplinary Research Group, Szeged, Hungary; Vrije Universiteit Brussel, BELGIUM

## Abstract

**Objective:**

Biomedical investment trends in 2015 show a huge decrease of investment in gastroenterology. Since academic research usually provides the basis for industrial research and development (R&D), our aim was to understand research trends in the field of gastroenterology over the last 50 years and identify the most endangered areas.

**Methods:**

We searched for PubMed hits for gastrointestinal (GI) diseases for the 1965–2015 period. Overall, 1,554,325 articles were analyzed. Since pancreatology was identified as the most endangered field of research within gastroenterology, we carried out a detailed evaluation of research activity in pancreatology.

**Results:**

In 1965, among the major benign GI disorders, 51.9% of the research was performed on hepatitis, 25.7% on pancreatitis, 21.7% on upper GI diseases and only 0.7% on the lower GI disorders. Half a century later, in 2015, research on hepatitis and upper GI diseases had not changed significantly; however, studies on pancreatitis had dropped to 10.7%, while work on the lower GI disorders had risen to 23.4%. With regard to the malignant disorders (including liver, gastric, colon, pancreatic and oesophageal cancer), no such large-scale changes were observed in the last 50 years. Detailed analyses revealed that besides the drop in research activity in pancreatitis, there are serious problems with the quality of the studies as well. Only 6.8% of clinical trials on pancreatitis were registered and only 5.5% of these registered trials were multicentre and multinational (more than five centres and nations), i.e., the kind that provides the highest level of impact and evidence level.

**Conclusions:**

There has been a clear drop in research activity in pancreatitis. New international networks and far more academic R&D activities should be established in order to find the first therapy specifically for acute pancreatitis.

## Introduction

Global biomedical research funding has started decreasing in the 21^st^ century. The budget for the NIH, which is the largest contributor to biomedical research, has steadily dropped from 2003 [1–3]. Moreover, data on corporate investment trends published by the Biotechnology Industry Organization in February 2015 showed a general decrease (from $21 billion (2004–2008) to $17 billion (2009–2013)) in research investment in novel drug research and development (R&D) and drug improvement R&D [4]. However, investment trends in the different disease categories (cardiovascular, immunology, gastroenterology, etc.) have been highly variable. Shockingly, the biggest drop was in the area of gastroenterology disease (62% from $828 million to $311 million), a wake-up call to academic researchers to boost research activity in the field. Since academic research usually provides the basis for industrial R&D, our aim was to understand the research trends in the field of gastroenterology and highlight the most endangered areas.

## Materials and Methods

### Analyzing scientific activity in the different areas of gastroenterology

In the first part of the study, we searched PubMed hits between 1965 and 2015 for pancreatic diseases (diabetes, pancreatitis and pancreatic cancer); benign GI diseases, such as upper GI tract disorders (reflux, oeseophagitis, Barrett’s syndrome and gastritis), lower GI tract diseases (inflammatory bowel diseases and irritable bowel syndrome) and hepatitis; and malignant GI diseases, such as gastric, oesophageal, colon, liver and pancreatic cancers. Altogether, 1,554,325 articles were analyzed.

### Detailed analyses of basic and clinical studies on pancreatitis and pancreatic cancer

Since the biggest drop in research activity was in pancreatology, in the second part of the study we aimed to search PubMed for ‘experimental pancreatitis’ (E-P; 3,767 articles were found), ‘experimental pancreatic cancer’ (E-PC; 3,697 articles), ‘pancreatitis AND clinical trial’ (C-P; 2,470), ‘pancreatic cancer AND clinical trial’ (C-PC; 4,321). Altogether, 14,255 articles were analyzed. All the available abstracts were checked. The final analyses were only performed on articles which contained original data in pancreatic research (6,628) in the categories described above. After the exclusions, we conducted a detailed analysis of 1,871 articles in E-P, 1,726 in E-PC, 1,079 in C-P and 1,952 in C-PC. The following parameters were collected from the articles: (1) number of countries and (2) number of centres involved in the research, (3) the journal’s impact factor (IF; based on the last available IF for the journal) and (4) whether the trial was registered in an official trial registry (only for clinical trials). An article was defined as ‘multinational’ if more than five countries were involved in the study and ‘multicentre’ if more than five centres took part. Analyses were performed for individual countries. An analysis of the individual parameters was conducted on the group of articles where the given parameter was available.

All PubMed searches took place on 23 December 2015.

### Limitations

The search was performed in PubMed, which provides a substantial selection of scientific literature, but of course it does not provide full coverage of all scientific activity. Another limitation was the lack of information on specific parameters in some of the individual abstracts. These abstracts were excluded from the analysis of that particular parameter. Finally, due to the extremely high number of articles, the impact factors (IF) of the articles were not calculated for the year of publication, but based on the journal’s IF for the most recent year (2014).

### Statistical analysis

To investigate differences in research activity, we compared the confidence intervals (CI) of the proportions. We used the equation for large samples,

$p^{*}\pm z\times \sqrt{\frac{p^{*}\times (1-p^{*})}{n}}$, where $p^{*}=\frac{m}{n}$, m = number of articles/disease and n = number of all articles.

To analyze the changes of research activity, we compared the slopes of the regression with an estimation of CI. One-way ANOVA was used with Dunnett’s post hoc test (unequal variances were assumed) to compare the IF between countries and centres. Chi-square tests were employed for relationship analysis. Statistical analyses were done by IBM SPSS Statistics v 20.0 (IBM Corporation, Armonk, NY, USA).

Values are expressed as means ± standard error (S.E.M.) if not stated otherwise. A p value <0.05 was considered statistically significant.

Data availability: Original data are available as supplementary materials (S1 and S2 Data).

## Results

### Research activity on pancreatitis has decreased compared to other gastrointestinal diseases

**Fig 1:**
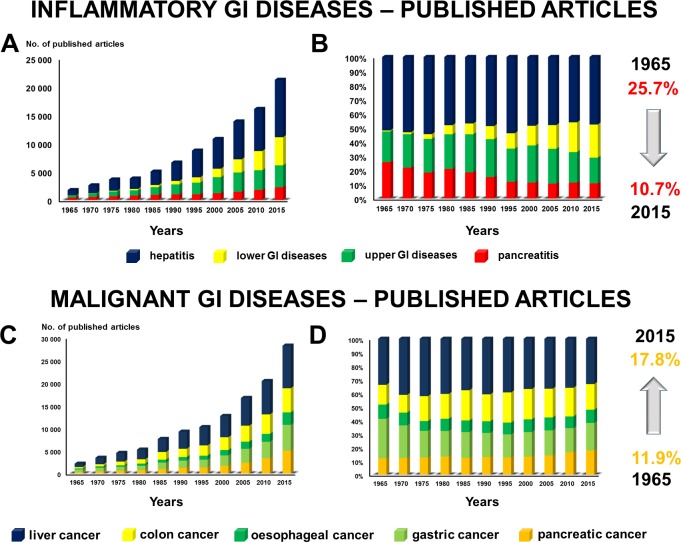
**A–B. Inflammatory GI diseases.** From 1965 to 2015, the great loss of interest in pancreatology was accompanied by a major increase of research in the lower GI disorders, namely, the IBD and IBS. **C–D. Malignant GI diseases.** The biggest increase was found in research activity on pancreatic cancer.

In the first part of the study, we characterized research activity on different parts of the GI tract. In 1965, among the major benign GI disorders, 51.9% (CI 49.58–54.22) of the research was performed on hepatitis, 25.7% (CI 23.63–27.75) on pancreatitis, 21.7% (CI 19.76–23.30) on upper GI diseases and only 0.7% (CI 0.34–1.13) on the lower GI disorders. Half a century later, in 2015, twelve times more research was being carried out on benign GI disorders. However, while research on the lower GI tract had increased 383 times, that on hepatitis eleven times and that on the upper GI tract ten times, the number of studies on pancreatitis had risen only five times. These nonparallel changes led to a situation in which only 10.7% (CI 10.27–11.11) of the research activity in 2015 was being performed on pancreatitis from among the benign GI disorders (Fig 1A and 1B). Since research on the upper GI tract and hepatitis rose parallel to the average increase of the research on the GI diseases, we can assume that the great loss of interest in pancreatology was accompanied by a great upturn in research in the lower GI disorders, namely, the IBD and IBS.

### Research activity on pancreatic cancer has risen slightly compared to other GI cancers

In 1965, among the major malignant GI disorders, research was conducted on the different forms of cancer as follows: cancer of the liver: 33.9% (CI 31.89–35.93); the stomach: 29.1% (CI 27.18–31.04); the colon: 14.6% (CI 13.05–16.05); the pancreas: 11.9% (CI 10.55–13.29); and the oesophagus: 10.5% (CI 9.20–11.80). Fifty years later, in 2015, twelve times more research was being performed on malignant GI disorders, an increase of exactly the same level as that of the studies on the benign GI disorders. While the relative research activity on liver and oesophageal cancer did not change, a clear decrease was observed in studies on gastric cancer (from 29.1% to 20.2%), with the biggest rise found in the research on pancreatic cancer (1.5 times) (Fig 1C and 1D).

### Research activity on pancreatitis has decreased compared to that on other major pancreatic disorders

**Fig 2:**
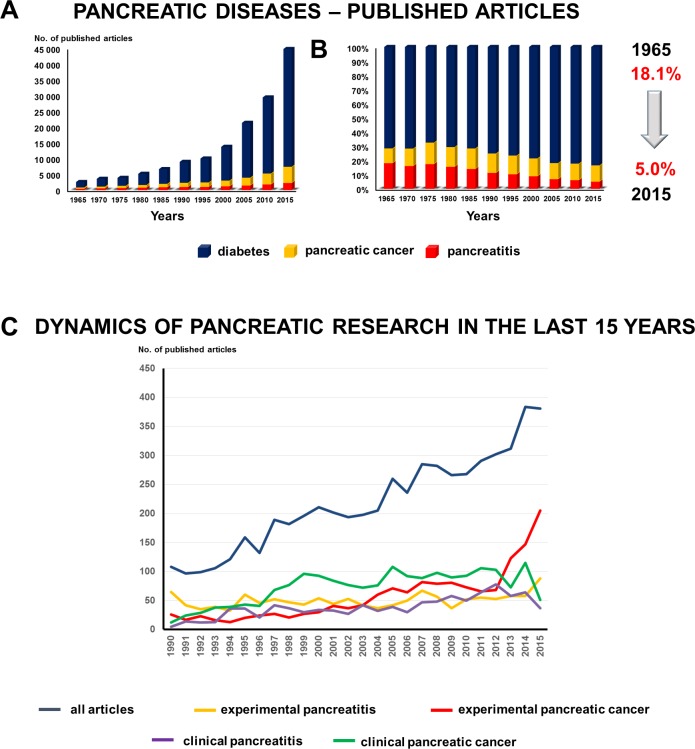
**A–B. Pancreatic diseases.** The relative interest in pancreatitis dropped from 18.1% to 5%. **C. Dynamic of pancreatic research.** The biggest rise of pancreatic research activity in the last five years was in experimental pancreatic cancer. However, the number of clinical trials–especially on pancreatitis–started decreasing.

Since the biggest drop in GI research interest was in the area of pancreatitis, we continued our study by analyzing the trends in pancreatic diseases. Here we compared the changes of research activity in diabetes, pancreatitis and pancreatic cancer. In 1965, 71.8% (CI 69.99–73.51) of the research was performed on diabetes, 18.1% (CI 16.63–19.65) on pancreatitis and 10.1% (CI 8.93–11.29) on pancreatic cancer. Although 18 times more studies were being conducted on the pancreas 50 years later, the relative interest in pancreatitis had dropped to 5% (CI 4.88–5.28). The relative activity did not change very much in pancreatic cancer (from 10.1 to 11.2%); however, research interest in the endocrine pancreas rose by 11.9% (Fig 2A and 2B). Analyzing the dynamic of the changes, we can assume that the biggest rise in pancreatic research activity in the last five years was in experimental pancreatic cancer. However, the number of clinical trials–especially on pancreatitis–started decreasing (Fig 2C).

### The USA, Germany and Japan publish the highest number of articles in pancreatology

**Fig 3:**
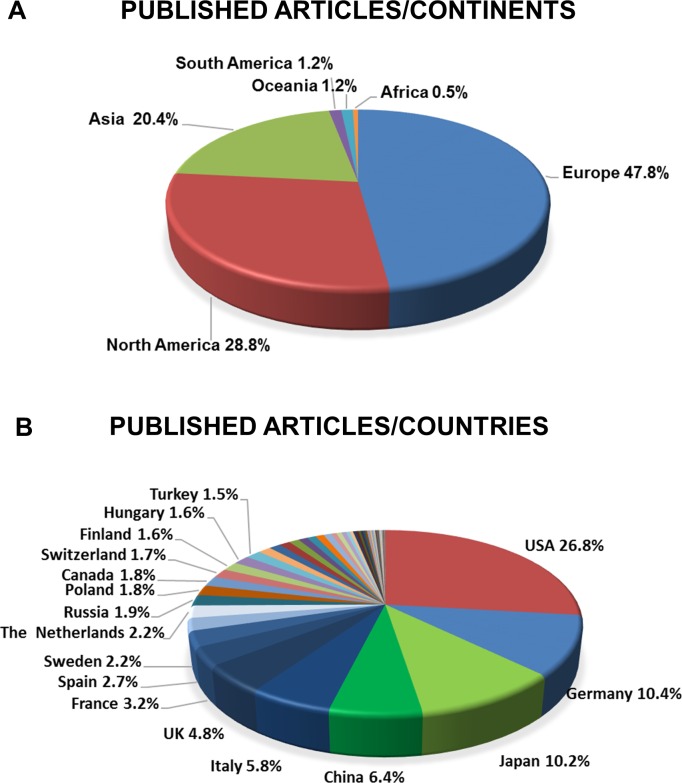
**A. Published articles per continent.** 47.8% of all the articles came from Europe and 28.8% from North America. **B. Published articles per country.** The USA, Germany, Japan and China together account for more than 50% of all published articles in pancreatology.

As stated above, 6,628 articles contained original research on basic or clinical pancreatology (involving 7,927 countries). As regards the continents, 47.8% of all participation involved Europe, 28.8% North America, 20.4% Asia and the Middle East, 1.2% Australia and Oceania, 1.2% South America and 0.5% Africa (Fig 3A). In terms of the four subgroups (E-P, E-PC, C-P and C-PC), while Europe has the leading role in E-P, C-P and C-PC studies, North America has the highest share in E-PC research. Among the subgroups, C-P has the lowest proportion of all articles on all the continents (S1 Fig).

**Fig 4:**
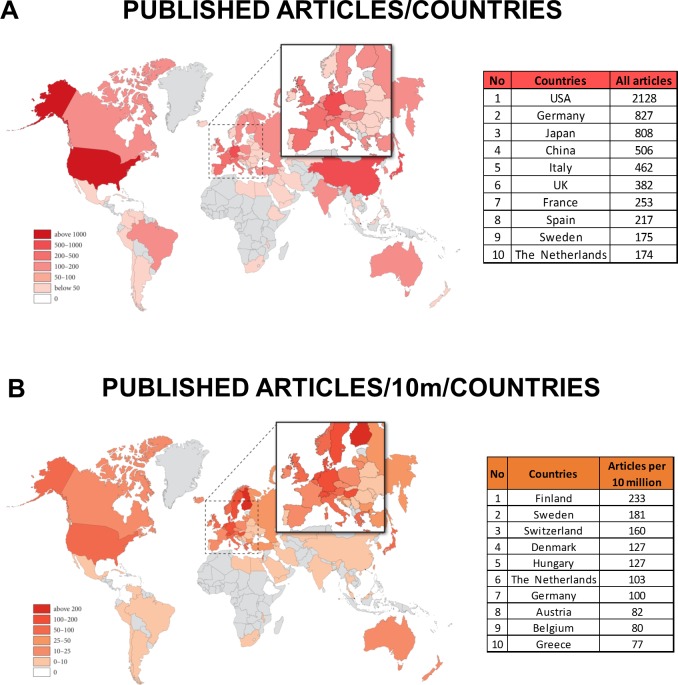
**A. Map of published articles.** The USA was involved in the largest number of research articles, followed by Germany, Japan and China. **B. Map of published articles per population.** The Scandinavian countries are clearly the most active in pancreatic research per capita.

With regard to the location of research, not surprisingly, countries with the largest population had an advantage: the USA was involved in the largest number of research articles (26.8%), followed by Germany (10.4%), Japan (10.2%) and China (6.4%) (Fig 3B and Fig 4A). Altogether, these four countries participated in more than 50% of the research on pancreatology. Detailed analyses of the four subgroups revealed that the USA led all four subgroups. The countries that ranked second in the subgroups were Germany in the experimental research groups (E-P and E-PC), China in C-P and Japan in C-PC (S2 Fig).

### The density of active pancreatic researchers is highest in the Scandinavian countries

Comparing the data per population of 10 million, small countries came to the fore. Scandinavian countries are clearly the most active in pancreatic research per capita. None of the big countries were in the top five (Fig 4B). Detailed analysis has also revealed interesting differences between the countries (S3 Fig). E-P research is led by Finland, E-PC by Switzerland, C-P by Denmark and C-PC by Sweden.

### The USA and the Netherlands are in the forefront in registered clinical trials

**Fig 5:**
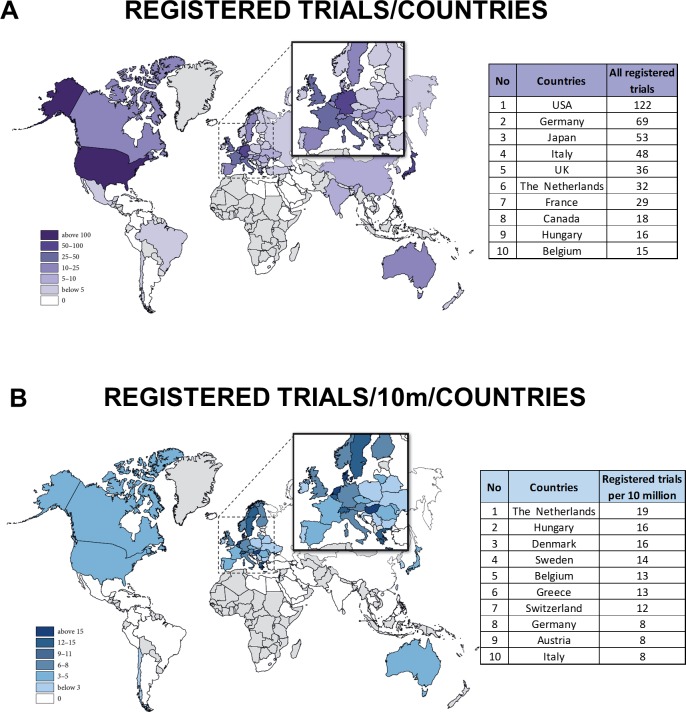
**A. Map of registered trials.** The big countries hold clear leading positions. **B. Map of registered trials per population.** Comparing the registered clinical trials per population of 10 million, Dutch researchers are the most active.

The highest level of evidence is obtained from registered clinical trials. With regard to the absolute numbers of registered clinical trials in pancreatology, the big countries register the highest number of trials (Fig 5A). Comparing registered clinical trials per population of 10 million, Dutch researchers are the most active (Fig 5B). Only 13.4% of all trials were registered.

### Multinational and multicentre studies provide the most valuable research in pancreatology

**Fig 6:**
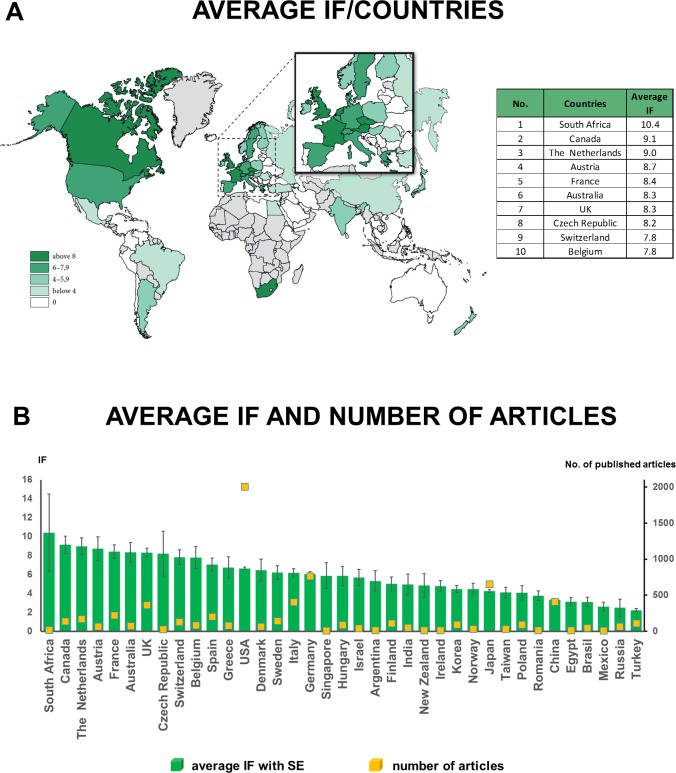
**A. Map of average impact factor/country**. There are no big differences between the average IF/country. **B. Average impact factor per country.** Over 30 countries achieved an average IF higher than 5. Values are expressed as means ± standard error (S.E.M.).

**Fig 7:**
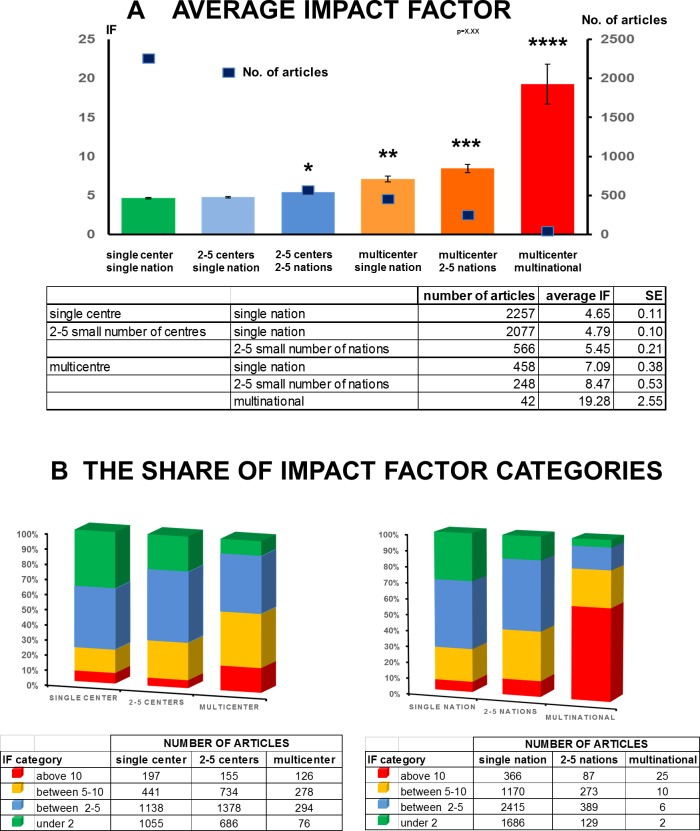
**A. Average impact factor by number of centres and nations.** Both multicentre and multinational approaches increase the impact of the papers. *: p = 0.009 vs. single centre single nation; **: p˂0.001 vs single centre/single nation; ***: p˂0.001 vs 2–5 centres/2–5 nations and vs multicentre/single nation; ****: p˂0.001 vs all groups. Values are expressed as means ± standard error (S.E.M.) **B. The share of average impact factor categories.** There is a strong correlation between the number of countries per study and the quality of the article.

Detailed analyses showed that there are no big differences between the average impact factors (IF) of countries. Countries with a low number of articles, such as South Africa and Canada, have the highest average IF. Over 30 countries produced an average IF higher than 5 (Fig 6A and 6B). Therefore, practically speaking, the quality of research is not country-dependent. However, detailed analysis of the articles revealed that there is a strong correlation between the number of countries per study and the quality of the article. In a single-nation article, the average IF is 4.652 (± 0.10), when only a single centre is involved. However, the involvement of more than six centres in a single nation increased the average IF of articles to 7.094 (± 0.37). Notably, multicentre and multinational studies achieved the highest average impact 19.278 (± 2.55) (Fig 7A and 7B).

## Discussion

With regard to gastrointestinal diseases, there is significant morbidity, mortality and, of course, spending within national health budgets [5–9]. In the USA, not only are 60–70 million people affected by such diseases each year, but they also cause around a quarter million deaths annually and generate an estimated cost of $150 billion per year [7]. There is no specific therapy for many of these diseases, including pancreatitis and pancreatic cancer [10–12]. Of course, first, the pathomechanisms of the disease should be understood, new therapeutic targets revealed and the biomedical industry attracted [13–16].

The levels of research activity in academia and the biomedical industry are remarkably interdependent [17–19]. Gastroenterology is no longer attractive for investment by biomedical firms or medical grant agencies [4]. Therefore, here we aimed to hold a mirror up to the researchers and funding agencies to better understand the research activity in the field. Of course, the hospitalization dynamics and requirements for different diseases in the areas of gastroenterology differ. Since 2000, while some hospital admissions have decreased for certain diseases (e.g. cholelithiasis by 14%, oesophageal reflux by 32% and alcoholic liver diseases by 5%) and others have risen (acute pancreatitis by 30%, clostridium difficile infection by 237%), the highest number of admissions are due to acute pancreatitis (over 250,000/year), with the highest annual costs ($2.5 billion) in the USA [7]. A Scottish study revealed a ten‐fold rise in the incidence of acute pancreatitis among men, and about half among women, from 1961 to 1985 [20]. After that, in the following ten years, it further increased around 65% [21]. Moreover, the incidence rate of chronic pancreatitis also rose. For example, within ten years, the hospital admission rate for chronic pancreatitis doubled in the UK [22].

Therefore, needless to say, boosting research activity in the field of pancreatitis is not only important medically but also economically. However, despite its great importance, pancreatic research suffered the biggest loss of interest in gastroenterology, a trend which could be either due to the lower activity in academic research and/or the lack of a specific therapy (i.e. no income for the companies) for most of the diseases affecting either the endocrine or the exocrine pancreas.

### What did we find and what can we do?

#### Strengths

It is clear from our analysis that both large and small countries are contributing to pancreatic research. The literature on pancreatology is dominated by the United States, Germany, China, Japan, Italy and the UK, just like in other scientific fields, such as ‘pain’ [23] and ‘oncology’ [24], whereas the density of pancreatic research is the highest in the Netherlands, Finland, Sweden, Denmark and Hungary. We have observed a positive trend in the publication of pancreatic cancer research, although the reason is definitely the multifactorial action plans, such as those in the USA and Europe (www.pancan.org, www.eupancreas.com), which increase awareness and may influence decision makers and promote grant funding [25].

#### Weaknesses

There are 50 countries in Europe, but only 23 are actively publishing in the field (with more than ten published articles each in 50 years). The majority (84.8%) of the articles under analysis represent a single nation, and 39.9% are single-nation and single-centre studies with no cooperation with others. Not surprisingly, without cooperation, the possibilities for data collection were limited; therefore, only a few high-quality multinational and multicentre observational clinical trials or RCTs were performed [26–31]. It is important to highlight that the Central and Eastern European, African, South American and Asian countries are facing the biggest difficulties as their sometimes poor infrastructure and lack of resources make them an undesirable research partner. Moreover, grant proposals submitted from these countries are usually rejected. More than 50% of the European countries (representing more than 200 million people!) are only slightly involved in pancreatic research, a situation which is a huge mistake and luxury in the field. In addition, patient care is also diminished since evidence-based guidelines are only published in a few countries in Eastern and Central Europe [32–37].

#### Opportunities

This analysis provides clear evidence that multicentre, multinational cooperation can achieve better-quality trials and higher impact in the field. International patient registries and biobanks should be created to stimulate quality multicentre observational trials, RCTs and translational research [38–44]. Importantly, following the success of pancreatic cancer action plans that probably contributed to the four-fold rise of E-PC research activity in the last few years, the same action should be initiated for pancreatitis.

#### Threats

If research on pancreatitis is to decrease further, journal editors may consider pancreatology an even lower priority, thus resulting in fewer publications in top journals. Perhaps it almost goes without saying that this will be followed by fewer grants and less activity in the field, thus continuing the vicious circle seen in the last 50 years, which has resulted in no specific treatment for acute pancreatitis.

## Conclusion

Substantially more academic research should be performed in gastroenterology. Activity in pancreatitis research has been rapidly decreasing. These data strongly suggest to funding agencies that they should consider pancreatitis an endangered field of research and sponsor far more international networks and academic R&D activities.

## Supporting Information

S1 Fig Published articles. **The share of the continents in the four research subgroups.** Europe has the leading role in E-P, C-P and C-PC, in the E-PC research North America has the highest share. Among the subgroups C-P has the lowest share of all articles in all continents.
(TIF)

S2 Fig Published articles. **The share of the countries in the four research subgroups.** The USA led all of the four research subgroups, however, the second was Germany in the experimental research groups (E-P, E-PC), whereas China had the second place in the C-P while Japan in the C-PC group.
(TIF)

S3 Fig Published articles per 10 million population in the four research subgroups. When we normalize the number of published articles to 10 M population, E-P research is led by Finland, E-PC by Switzerland, C-P by Denmark and C-PC by Sweden.
(TIF)

S1 Data Raw data by countries. (XLSX)

S2 Data Analysis and Figures. (XLSX)
