# Supplementary figures and images for: Analysis of Research Activity in Gastroenterology: Pancreatitis Is in Real Danger
Source: PLoS One. 2016 Oct 24;11(10):e0165244. doi: 10.1371/journal.pone.0165244 (PMC5077088; doi:10.1371/journal.pone.0165244)

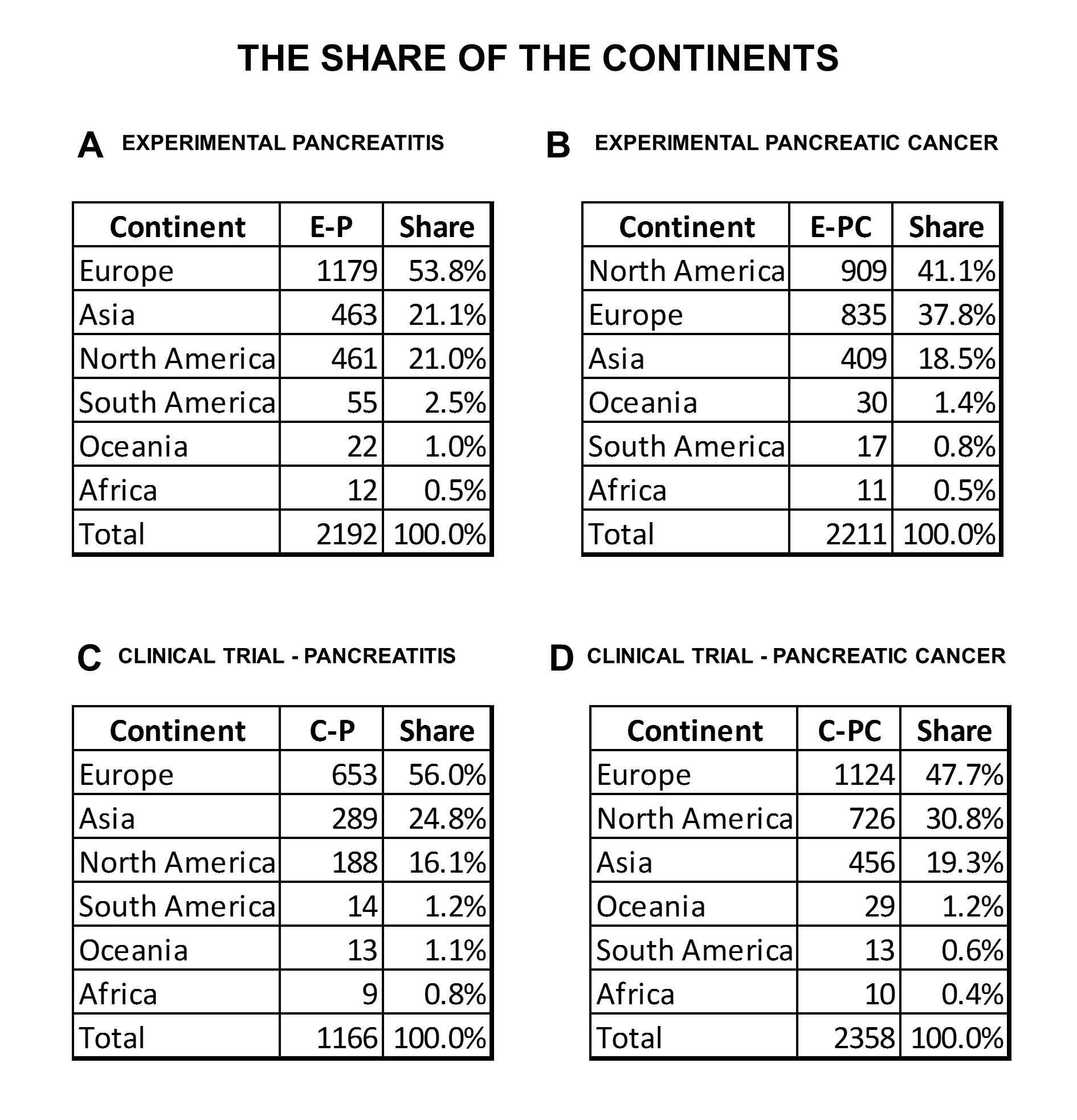

Supplement: S1 Fig — The share of the continents in the four research subgroups. Europe has the leading role in E-P, C-P and C-PC, in the E-PC research North America has the highest share. Among the subgroups C-P has the lowest share of all articles in all continents. (TIF) [file pone.0165244.s001.tif]

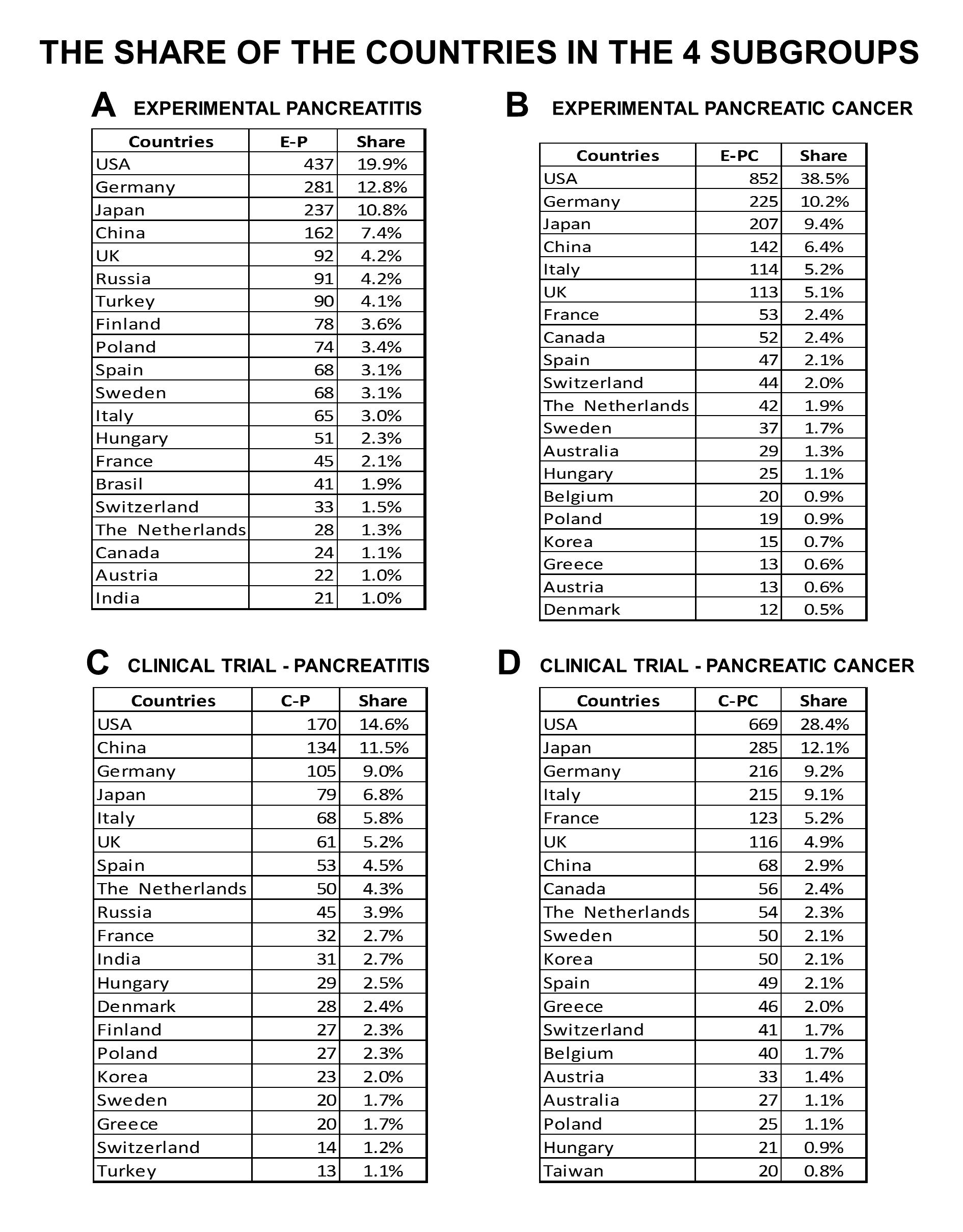

Supplement: S2 Fig — The share of the countries in the four research subgroups. The USA led all of the four research subgroups, however, the second was Germany in the experimental research groups (E-P, E-PC), whereas China had the second place in the C-P while Japan in the C-PC group. (TIF) [file pone.0165244.s002.tif]

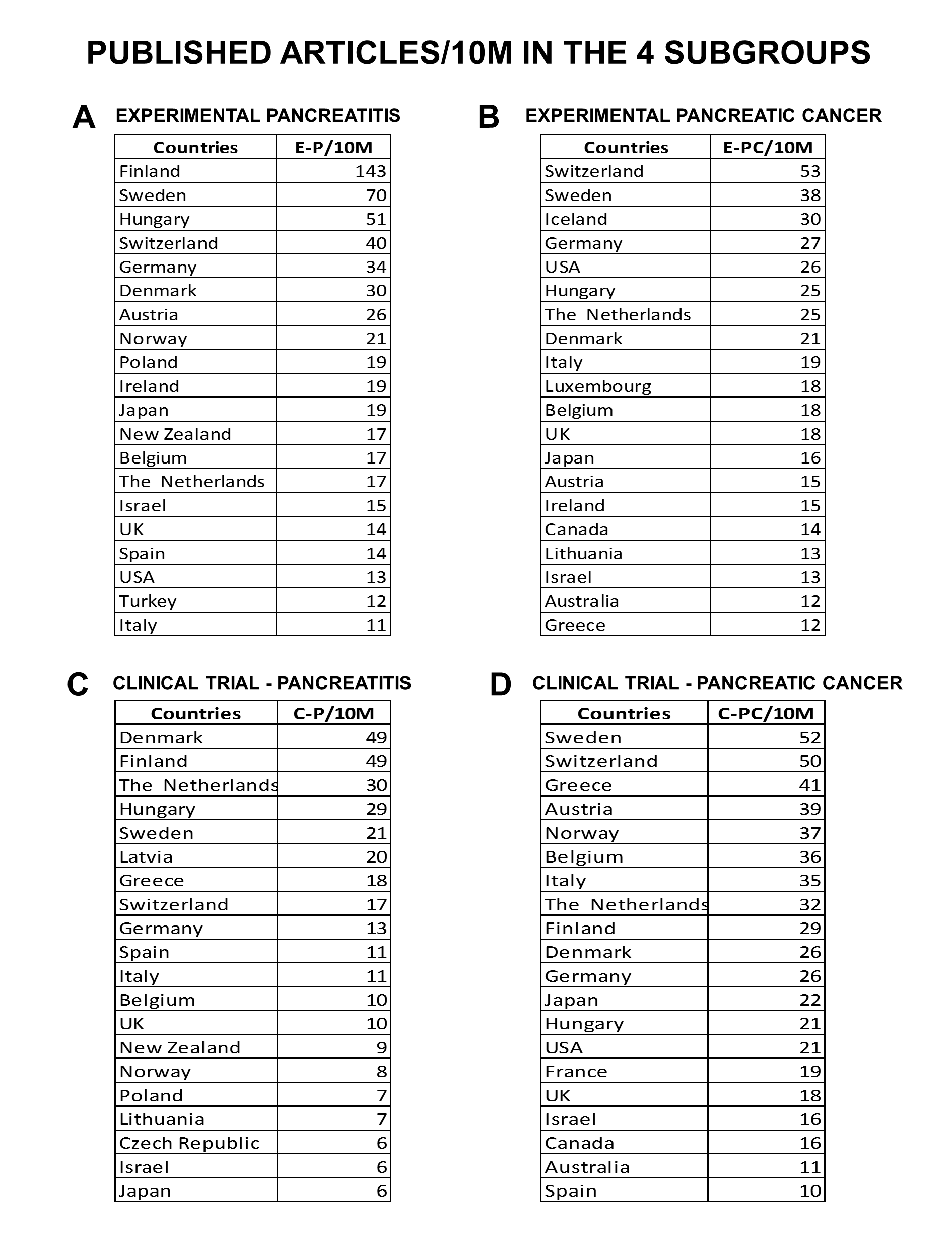

Supplement: S3 Fig — When we normalize the number of published articles to 10 M population, E-P research is led by Finland, E-PC by Switzerland, C-P by Denmark and C-PC by Sweden. (TIF) [file pone.0165244.s003.tif]
